# Practices and Challenges of Veterinary Paraprofessionals in Regards to Antimicrobial Use and Resistance in Animals in Dar Es Salaam, Tanzania

**DOI:** 10.3390/antibiotics10060733

**Published:** 2021-06-17

**Authors:** Gasto Frumence, Leonard E. G. Mboera, Calvin Sindato, Anna Durrance-Bagale, Anne-Sophie Jung, Stephen E. Mshana, Taane G. Clark, Helena Legido-Quigley, Mecky I. Matee

**Affiliations:** 1 Department of Development Studies, School of Public Health and Social Sciences, Muhimbili University of Health and Allied Sciences, P.O. Box 65454, Dar es Salaam 65001, Tanzania; 2 SACIDS Foundation for One Health, Sokoine University of Agriculture, Morogoro 3019, Tanzania; lmboera@gmail.com (L.E.G.M.); csindato@gmail.com (C.S.); stephen72mshana@gmail.com (S.E.M.); mateemecky@yahoo.com (M.I.M.); 3 National Institute for Medical Research, Tabora Research Centre, P.O. Box 482, 026 Boma Road, Tabora 45026, Tanzania; 4 London School of Hygiene and Tropical Medicine, London WC1H 9SH, UK; anna.durrance-bagale@lshtm.ac.uk (A.D.-B.); anne-sophie.jung@lshtm.ac.uk (A.-S.J.); taane.clark@lshtm.ac.uk (T.G.C.); ephhlq@nus.edu.sg (H.L.-Q.); 5 Department of Medical Microbiology, Catholic University of Health and Allied Sciences, P.O. Box 1464, Mwanza 33109, Tanzania; 6 Department of Microbiology and Immunology, School of Medicine, Muhimbili University of Health and Allied Sciences, P.O. Box 65001, Dar es Salaam 11103, Tanzania

**Keywords:** antimicrobial use, antimicrobial resistance, veterinary paraprofessionals, perception, Tanzania

## Abstract

We conducted a qualitative study to explore the practices and challenges of veterinary paraprofessionals (paravets) on antimicrobial use and resistance in domestic animals. Methods: This was a qualitative study, which involved semi-structured interviews with paravets from the Ilala, Ubungo, Kigamboni, Kinondoni, and Temeke districts in Dar es Salaam, Tanzania. Results: A total of 40 paravets participated in this study. The majority (72.5%) admitted to having not undergone any formal training on antimicrobial use and/or resistance. Paravets face several challenges, including poor working conditions and having no access to laboratory services to advise on antimicrobial choice and selection. They also face challenges from livestock farmers such as the inability to afford the recommended medicines, the self-prescription of antimicrobials, and poor record keeping. The presence of sub-standard medicine and the lack of guidelines on the appropriate disposal of medicines were also identified as affecting their services. Conclusion: Paravets should be trained in the judicious use of antimicrobials, and the same training should be used to refresh their knowledge on the diagnosis and prevention of infections. The Veterinary Council of Tanzania and other regulatory agencies should assist in addressing the challenges facing paravets that are related to animal health services and the quality of medicines.

## 1. Introduction

Tanzania is among the leading African countries in livestock production, second only to Sudan and Ethiopia in the number of domestic livestock [1]. The country is estimated to have 21.3 million cattle, 13.1 million goats, 3.6 million sheep, 1.5 million pigs, and over 30 million poultry [2]. Almost all animals (99%) are owned and managed by small-scale farmers and pastoralists. Livestock provide direct livelihood support to about five million households [3]. The country’s demand for meat is high and increasing, which has led to intensive animal production [4,5,6,7]. 

It was estimated that the annual chicken meat and domestic pig production would increase by about 258% from 130,000 tons in 2017 to 465,600 tons in 2020, and by 69% from 22,000 in 2017 to 37,200 tons in 2022, respectively [8]. This increase has been attributed to a number of factors including the increasing urbanization rate, resulting in the increased trade of live animals and animal products [7]. Such large numbers of livestock, as well as the projected increase, require the proper management of livestock and the provision of good, accessible, and affordable veterinary services. However, this remains grossly inadequate in many countries in Africa [9]. In Tanzania, livestock production has been associated with several challenges, including overstretched veterinary extension services and the lack of implementation of disease control strategies [10,11,12,13,14,15]. Not surprisingly, despite accounting for 11% of the African cattle population, livestock production contributes only 7.4% to Tanzania’s gross domestic product (GDP), and growth of the livestock sector, at 2.6%, is low [8]. This growth largely reflects increases in livestock numbers, rather than productivity gains, which are constrained by low livestock reproductive rates, high mortality, and high disease prevalence [8]. 

For most low- and middle-income countries (LMICs), animal healthcare systems are underfunded, and regulatory capacities to promote prudent antimicrobial use (AMU) and control antimicrobial resistance (AMR) are very limited [16,17,18]. Antimicrobials are sourced at local, privately owned agrovet drug shops, and knowledge; attitudes; and, particularly, practices towards using them vary greatly [19,20,21]. Often, farmers diagnose and treat their own animals, a practice that increases the likelihood of the misuse of drugs, wrong drug choice, incorrect routes of drug administration, inconsistent administration intervals, and a lack of observation of the withdrawal period [19,20,21,22,23]. In Tanzania, the supply chain of veterinary medicines from the sources to the end users is carried out by the private sector. The government is mainly involved in regulating and monitoring through the Tanzania Medicines and Medical Devices Authority (TMDA) and the Veterinary Council of Tanzania (VCT). Veterinary antimicrobial agents are imported predominantly from Europe and Asia [24]. Once imported, they are distributed to sellers and eventually to the end users of antimicrobial agents. With weak regulations, antimicrobial agents are commonly sold by informal vendors such as petty traders and even livestock keepers during auction markets for livestock [25]. Antimicrobial agents found in markets such as these are often unregistered, and are therefore sold at cheap prices and are convenient sources of medicines for paravets and farmers. Post-market surveillance is virtually non-existent [24]. 

Veterinary paraprofessionals (paravets), whose education varies greatly around the world both in duration and quality, have, for some time now, been considered one of the most promising means of increasing the provision of animal health services in LMICs, especially in rural areas [26]. Key features of paravets are their low overhead costs and their willingness to provide services to small-scale producers in areas that could not otherwise support a state or private veterinarian. Usually, paravets are able to provide relatively cheap, locally available basic animal health care services that can lead to quite dramatic improvements in herd health [27]. Moreover, most livestock farmers in Tanzania buy drugs to treat their animals from veterinary drug shops, which are mostly run by paravets.

In Tanzania, matters pertaining to the prevention and treatment of livestock diseases are the responsibility of the veterinarians. However, the limited number of qualified veterinarians has led paravets to provide services ranging from disease surveillance to artificial insemination, animal treatment, vaccinations, and extension services, among others, virtually without any adequate supervision. Under the Tanzania Veterinary Act of 2003, paravets are certificate or diploma holders in animal health, recognized by the Veterinary Council of Tanzania (VCT), and practice at all times under the supervision of a veterinarian or veterinary specialist [28]. There are currently 1021 registered veterinarians and 4118 veterinary paraprofessionals in the country (Veterinary Council of Tanzania, unpublished report), which makes supervision of paravets’ work virtually impossible. Many studies indicate that most clients interact with paravets, and only a handful have ever encountered a veterinarian or veterinary specialist [29,30].

Inadequate veterinary services are also due to the fact that most of the veterinarians are located at the district level, where they often carry out administrative tasks. Thus, in most of the rural areas of Tanzania, paravets offer the only animal health care for the majority of farm animals. The need for paravets is also due to the inability of veterinarians to physically reach all of the animals, due to vastness of the country and the remoteness of some villages. It is documented that well-trained paravets, being close and easily accessible to the communities, provide an appropriate strategy for delivering affordable and sustainable animal health services, particularly where services are inadequate [29]. 

Since most of the animal health services, including diagnosis, treatment, and prophylaxis, in Tanzania are provided by the paravets, they are therefore likely to be involved in the use of antimicrobials. This group of animal health providers is important in strengthening antimicrobial stewardship. International organizations and governments have argued that animal health service providers can play an important role in promoting the prudent use of antimicrobials, thus limiting antimicrobial resistance [28]. However, in Tanzania, there is limited information regarding the practice and challenges of AMU and AMR in animal farming among paravets, who provide the bulk of animal health services in the country [31]. Their formal training curriculum indicates that minimal information is given on AMU, usually limited to prescribing a few antibiotics, mainly tetracylines, and there are no organized post-training seminars/workshops. We therefore conducted this study to provide insight into the practices and challenges of paravets, specifically on AMU and AMR in domestic animals. 

## 2. Results

This study involved semi-structured interviews with 40 Paravets (male: 70.0%, and female: 30.0%) from the Ilala, Ubungo, Kigamboni, Kinondoni, and Temeke districts in the Dar es Salaam region. Eight paravets were selected from each district, with half of them from the public and half from the private sectors. The majority (84.5%) of the study participants had a diploma level of education, with working experience ranging from 3 to 10 years. Almost three quarters (72.5%) had no training on AMU and/or AMR through refresher courses/seminars post-formal education (Table 1).

Analysis of the findings generated two main themes and several subthemes, which are presented in the following section. The first main theme is the practices and challenges of paravets in providing animal health care services. This theme has three sub-themes: (i) the lack of adequate training in AMU/AMR among paravets, (ii) the poor quality of medicines and working tools, and (iii) the availability and affordability of veterinary services. The second main theme is the clients’ understanding of AMU and AMR and the resulting behaviors, which has six subthemes: (i) clients’ low understanding of AMU and AMR, (ii) the self-prescription of antibiotics, (iii) livestock keepers’ preferences, (iv) the disposal of drugs and other waste; (v) awareness of the antimicrobial withdrawal period, and (vi) record keeping. 

### 2.1. Practices and Challenges of Veterinary Paraprofessionals in Providing Services

#### 2.1.1. Lack of Adequate Training of AMR/AMU in Veterinary Paraprofessionals

Most of the interviewed paravets reported that they have not attended refresher courses or seminars on AMU and AMR apart from the training that they attained when they were pursuing their formal education. It was reported that this situation has limited their understanding of AMU and AMR issues.

*“The truth is that I have never attended any training on AMU or AMR. There is one which I was informed but I was away in Morogoro and couldn’t attend.”* (KI number 5-Kigamboni).

Another paravet had this to say: *“I once attended a seminar in Dar es Salaam about livestock keeping especially on how to raise broiler chicken and also the use of medicine in domestic animals. However, I have never received training on the uses of antibiotics and bacteria resistance against antibiotics.”* (KI number 3-Kinondoni).

Some paraprofessionals claimed that they had received short courses, which were insufficient to equip them with the skills to address AMR.

*“I did not get any training. We only get partial training like two days’ session in a year when we met with veterinarians who tried to remind us on the use of antibiotics and the importance of the right treatment.”* (KI number 2-Kinondoni).

#### 2.1.2. Poor Quality of Medicines and Working Tools

Interviews revealed concerns regarding the veterinary medicines available in the market regarding multiple sources of importation. In addition, it was reported that the quality of drugs is largely unregulated, such that sub-standard and falsified medicines are commonly available on the market.

*“Lack of regulations has led to many livestock keepers to believe that some antibiotics from certain companies are more effective compared to other companies. For example, when you take tetracycline from two different companies you will find that the effectiveness is not the same, and this is reflected to the price. The most effective antibiotics are sold more expensive compared to less effective ones.”* (KI number 4-Ilala).

On the other hand, paravets argue that they do not have the ability to preserve veterinary medicines that require special storage conditions.

*“You find out that ambient temperature in our setting is 28 °C, and the drug manufacturer instruction requires keeping the drug in the room with air conditioner or fan. We frequently keep the drugs in the bag.”* (KI number 2-Ilala).

Our key informants also reported that they face difficulties in carrying out their activities because of limited working tools, including medical kits and means of transport.

*“The main challenge is lack of kits for carrying drugs and lack of transport. Recently we were carrying medicines in our bags. In terms of transport, even when I have own means of transport, I need money for fuel.”* (KI number 3-Kigamboni).

#### 2.1.3. Availability and Affordability of Veterinary Services 

The findings revealed that there are few paravets in the community, while the demand for their services is high. Furthermore, some villages are located in remote areas, which cannot be easily reached by the paraprofessionals.

*“There are areas not accessible by vehicle and/or motorcycle and if you dare to go you will have to ask someone to ferry you before you walk long distances. Animal health experts hesitate to go to such areas.”* (KI number 1-Ilala).

Most of the interviewed paravets reported the limited availability of veterinary medicines on the market (veterinary shops). This prompts them to most often use the drugs that are available, even if they are not effective.

*“The challenge is the unavailability of antibiotics or their cost. You find that sometimes the drugs are not available even at veterinary shops. When they are available, they are expensive and few farmers could afford.”* (KI number 3– Ilala).

*“Sometimes the antibiotic you need is not available so you use an alternative drug, regardless of its effectiveness.”* (KI number 1-Kigamboni).

It was reported that there are untrained individuals who provide veterinary services to livestock keepers. There are fake animal health providers in the community. They have neither received any formal training nor been registered by the government authorities, but they claim to have ‘experience’ in animal health.

*“May be the recently issued guidelines, which aim to address this prevailing challenge so that unregistered individuals will not be allowed, may solve the problem.”* (KI number 5-Ubungo).

Most of the clients (livestock keepers) cannot afford to purchase expensive antibiotics, so they will always opt for the cheapest ones. One paraprofessional had this to say:

*“Sometimes my clients would call me to attend their animals. Once I use a certain antibiotic of a certain company, they will tell me that they cannot afford the cost because they are expensive. So, I would be forced to opt for the drug which the client can pay the costs.”* (KI number 4-Kigamboni).

### 2.2. Clients’ Understandings of AMU and AMR and Resulting Behaviors

#### 2.2.1. Clients’ low Understanding of AMU and AMR

Interviewed paravets reported that their clients (livestock keepers) have little understanding of AMU and AMR, a situation that can be substantiated by non-adherence to the prescription given to them by paraprofessionals.

*“One of the major challenges is understanding. For example, I may advise a livestock keeper to use a certain medicine and he accepts. However, others would say “I don’t use that”. You cannot force him because the animals belong to the livestock keeper. In such situations we keep on educating them hoping for a change in attitude. This will however, take a long time.”* (KI-Kinondoni).

*“Another challenge is that some livestock keepers, like the Maasai have low understanding. Yet, sometimes they pretend that they know much better than paraprofessionals to the extent that they don’t adhere to instructions provided by the experts.”* (KI number 3-Ubungo).

#### 2.2.2. Self-Prescription of Antibiotics

Related to the livestock keepers’ lack of understanding of AMU and AMR, paravets further reported that the self-prescription of antibiotics is a common practice among livestock keepers. These antibiotics are mostly obtained over the counter without a prescription, and some of them are left over from previous treatments.

*“There is improper use of antibiotics, which is caused by self-prescription. It often happens that livestock keepers prescribe drugs for their animals especially those who live far from where veterinary services are available. They may call you to go and treat their sick animals but once you are late; they will buy the antibiotics and treat their animals. When you arrive, they will just inform you that you are late, so they have already administered the drug.”* (KI number 2-Kigamboni).

#### 2.2.3. Livestock Keepers’ Preferences 

Most of the interviewed paravets reported that livestock keepers’ preference of certain antibiotics is a common challenge.

*“The challenges are many. These include livestock keeper’s drug preference, little knowledge of animal husbandry. Thus, you may find that despite giving a lot of instructions, advices, and directions, they do not comply.”* (KI number 2-Temeke).

*“Some livestock keepers are dishonest because when they are given prescription of certain drugs to treat their animals they change and purchase different drugs of their preferences.”* (KI - Kinondoni).

*“Sometimes you find livestock keepers go against experts’ advice because they already have their own preferences or due to peer influence. Yet, sometimes by being misled by profit driven and advice from unprofessional drug sellers.”* (KI number 6-Temeke).

#### 2.2.4. Disposal of Drugs and other Wastes

Interviewed paravets reported that lack of regulations in regard to the disposal of expired drugs and drug waste is one of the most critical challenges that can lead to AMR.

*“Throwing wastes is another challenge especially for large livestock keepers; those having small land areas for animal keeping. Most of the animal wastes have antibiotics residuals, which are dangerous to human health and the environment.”* (KI number 4-Kinondoni).

#### 2.2.5. Awareness of Antimicrobial withdrawal Periods

According to the responses from the paravets, it was revealed that most of the livestock keepers do not comply with the antimicrobial withdrawal period.

*“I have never seen someone disposing eggs because they were produced when the chickens were taking antibiotics. Instead, they sell them. That is why even internationally our market is low because of low quality of eggs.”* (KI number 7-Kinondoni).

Some livestock keepers were reported to ignore the withdrawal period even when they knew about it, as this affects their business and profits:

*“Another challenge is dishonest from the livestock keeper. For example, you have used antibiotics, that restrict the use of milk for a week or two. But unfaithfully farmers wait for two or three days only and start to use and letting the milk to the market, which is likely to affect the health of consumer.”* (KI number 5-Kinondoni).

### 2.3. Record Keeping 

Interviewed paravets reported that livestock keepers need to keep the disease and treatment records because they are important components of good animal husbandry to keep track of the diseases which affect each animal in the herd throughout its lifetime. However, given their low level of understanding of AMU and AMR, record keeping regarding animal diseases and their treatment is not a common practice, as expressed by one of the key informants.

*“Livestock keepers do not keep treatment records. For example, when I visit my clients, I found that some of them are keeping records properly, thus I will advise them appropriately. However, other clients do not keep records making it difficult to advise them and treat their animals properly.”* (KI number 2-Kinondoni).

## 3. Discussion

The findings of this study revealed the importance of paravets not only in rural, but also in peri-urban and urban areas, where livestock keeping is associated with high AMU and AMR [19,22]. In these urban districts of Dar es Salaam, we found that paravets were providing a wide range of veterinary services to a variety of domestic animals. These services ranged from disease surveillance to artificial insemination, treatment, vaccinations, and extension services. The findings revealed that there are insufficient paravets in the community, while the demand for their services is high. On a positive note, the government of Tanzania recognizes the importance of this cadre, and they are registered with the VCT. Importantly, The Tanzania Livestock Master Plan [8], which lobbies for a significant increase in the number and quality of livestock, has outlined the roles of paravets in the communities that they serve, potentially bringing affordable veterinary services closer to them. It was encouraging to note that all the interviewed paravets were diploma holders and registered with the Veterinary Council of Tanzania.

We noted a number of significant challenges among paravets with regard to the management of AMU and AMR. It was striking to note that three-quarters of the interviewed veterinary paraprofessionals had not attended any formal training on AMU and AMR, and for the few who had attended training, it was mainly short seminars, hardly adequate to sufficiently inform livestock keepers on the judicious use of antimicrobial agents. This is very worrisome, since the inadequate knowledge of paravets on judicious AMU adversely influences their practice in terms of antimicrobial management [32,33]. 

We also noted that most paravets work under harsh conditions with limited support, being self-employed with unreliable income that depends on the availability of clients, who are mostly of low income [19,22]. As a consequence, most of them lack protective gear, access to diagnostic laboratories, and the ability to store essential medicines. Although the law requires paravets to work under the supervision of veterinary officers, this is hardly the case, and therefore a key concern remains regarding the quality of the services provided by paraprofessionals and the level of drug misuse that might arise through them. 

Some of the challenges faced by paravets pertain to the knowledge and practices of their clients. These include the self-prescription of antimicrobials, which appears to be a very common practice by most of the livestock keepers and has been associated with the improper use of human medicines for treating animal infections [23]. In addition, paravets have to contend with livestock keepers’ preferences, such as the choice of medication, even though clients may have little knowledge of animal health, with a high possibility of misdiagnosis, improper dosage, and the wrong choice of medicine [19,22,34,35,36,37]. Peer influence has also been reported to influence the choice of medication, as well as being misled by profit-driven and unprofessional drug sellers [38]. People who have no training in pharmaceutical products sell most of the veterinary medicines, and they have no knowledge with which to advise farmers [22]. Furthermore, during the interviews, it became apparent that most of the livestock keepers own small-scale animal herds, and cannot afford to purchase expensive antimicrobials, instead, they will always go for the cheap ones, which cannot yield effective treatment, thus leading to drug resistance. The interviewees acknowledged that most of the livestock keepers do not comply with the antimicrobial withdrawal period, either due to lack of awareness or motivated by profit, a finding which has been reported by others [22,37]. In addition, our findings revealed that record keeping among livestock keepers is a big challenge for the majority of farmers, which is important for keeping track of diseases and treatment outcomes. 

Several regulatory issues were also unveiled. The paravets revealed concerns regarding the veterinary medicines available on the market regarding multiple sources of importation, unregulated quality checks, and sub-standard or falsified medicine. Logically, farmers resort to buying the less expensive medicines, and usually over the counter, fueling the possibility of AMR [39,40]. The paravets reported a lack of regulations on the disposal of expired drugs and waste, giving rise to the contamination of the environment with resistant organisms and the possibility of spilling over to humans and animals [22,41]. Of particular concern is the fact that there are unregistered animal health practitioners in the community, who have no formal training and benefit from unsuspecting clients, which should alert the Veterinary Council of Tanzania and the Government to take stern measures. 

## 4. Methodology

### 4.1. Study Design

This qualitative study was conducted in five districts of the Dar es Salaam region: Ilala, Ubungo, Kigamboni, Kinondoni, and Temeke. We conducted in-depth interviews with paravets from the five districts. 

### 4.2. Study Settings

Dar es Salaam is the largest city in Tanzania, characterized by a high rate of urbanization (rural-urban migration) coupled with the growth of spontaneous settlements. It is the region with the highest population density of humans (3133 humans/square kilometer) [42] and chickens (918 chickens/square kilometer) [43]. It is characterized by high fishing industry and commercial activities, and remains as one of the largest destinations for livestock and livestock products from almost all areas of the country. The region harbors the Msimbazi river basin, characterized by intensive agricultural and farming practices involving the use of manures, pesticides, and antimicrobial agents, and is polluted with effluents and waste from the largest pharmaceutical and commercial industries in the country [44,45,46]. We assumed that the consumption of livestock and their products is high in areas with a high human population. Accordingly, the increased demand for livestock and their products is assumed to translate to increased production intensity and demand for veterinary services, as well as high antimicrobial use (AMU). The practices of inappropriate AMU in the region have been reported to be high [19,22]. Given the fact that increased AMU is associated with the increased risk of antimicrobial resistance (AMR), the Dar es Salaam region was considered to be an ideal site for this study to explore the experiences of veterinary paraprofessionals regarding AMU, and to estimate the risk of AMR.

### 4.3. Selection and Recruitment of Study Participants

The study employed a purposive and snowball sampling strategy to draw the key informants from all five districts. We identified key informants through a mapping exercise and under the guidance of an official from the districts and wards. Paravets were purposively selected because of their role in the delivery of animal health services. We stopped recruiting when it was jointly agreed that thematic saturation had been achieved—i.e., there were fewer surprises in the data and no more new codes, themes, or patterns emerged.

### 4.4. Data Collection, Management, and Analysis

This study adopted an interpretive approach to focus on the ways in which interviewees understood and made sense of the topics discussed. We employed semi-structured interviews to collect data from selected paravets. Interviews lasted between 45 and 90 minutes, and were run by 14 research assistants. All research assistants were trained according to the Guidelines on Ethics for Health Research in Tanzania [47]. All interviews were transcribed verbatim and translated from Kiswahili to English. We used a thematic data analysis approach and applied both inductive and deductive reasoning [48]. All investigators collectively identified and validated the emerging themes across a sample of the transcripts before a line-by-line analysis was conducted by the first author using NVivo 12 qualitative data analysis software (QSR International Pty Ltd. Version 12, 2018, Los Angeles, CA, USA). The emerging themes were searched, developed, and categorized into eight thematic areas. Each excerpt includes the number of the interview, code letters (KI for key informant), and the study setting, so that extracts from the same individual can be linked.

### 4.5. Ethical Considerations

All study participants provided their informed consent for inclusion before they participated in the study, and they were informed about anonymity and confidentiality issues and that they could withdraw from the study at any time they wished. The Medical Research Coordinating Committee of the Tanzania National Institute for Medical Research approved the protocol (Ref. No. NIMR/HQ/R.8a/Vol IX/3147). The study was conducted following the principles of the Declaration of Helsinki.

## 5. Conclusions

Our findings indicate that a large proportion of paravets have no formal training on AMU and AMR. We therefore recommend that the ministry responsible for livestock development and the Veterinary Council of Tanzania organize short courses on judicious AMU and the consequences of AMR for practicing paravets. We also propose a special training program on antimicrobial agents and resistance for veterinary paraprofessionals who are now undergoing their studies as part of their curriculum. We are also recommending that veterinarians and veterinary specialists should play a greater role in the daily supervision of paravets. There is also a need to enforce the regulations on the quality control of medicines, and conduct point of sale inspections. Finally, based on our results, we suggest that livestock keepers may benefit from more training on awareness and understanding of the implications of AMU and AMR.

## Figures and Tables

**Table 1:** Key characteristics of study participants.

Characteristic		Number	%
Sex	Male	28	70.0
	Female	12	30.0
Sector	Public	20	50.0
	Private	20	50.0
Level of education	Diploma	35	87.5
	Certificate	5	12.5
Work experience (years)	≤10	17	42.5
	11–20	12	30
	≥21	11	27.5
Training on AMU/AMR	Yes	11	27.5
	No	29	72.5

## Data Availability

The data presented in this study are available on request from the corresponding author.
